# Supplementary material for: Expertise-dependent differences in mental representation metrics of pas de bourrée
Source: PLoS One. 2023 Oct 5;18(10):e0292133. doi: 10.1371/journal.pone.0292133 (PMC10553204; doi:10.1371/journal.pone.0292133)
Supplement: S2 File — Includes more detailed information regarding gender, age and training experience of the subjects. (PDF) [file pone.0292133.s002.pdf]

Overview of the subjects

Advanced group

LUBI09
6 years, 3x week:
50 x 6 x 3 = **900**
Advanced
Female, 24 years

RUMA29
14 years, 2x week:
14 x 50 x 2 = **1400**
Advanced
Female, 23 years

RISI10
20 years, 2x week
20 x 50 x 2 = **2000**
Advanced
Female, 24 years

RNHE11
21 years, 2x week
21 x 50 x 2 = **2100**
Advanced
Female, 27 years

TAOE21
24 years, 2x week
24 x 50 x 2 = **2400**
Advanced
Female, 28 years

REHE20
15 years, 2x week
15 x 50 x 2 = **1500**
Advanced
Female, 21 years

**Gender**	**Male**	**Female**		
	-	6x		
**Experience in their sport**	**5–10 years**	**10–15 years**	**15–20 years**	**>20 years**
	1x	2x	1x	2x
**Training sessions per week in their sport**	**0–2**	**3–4**		
	4x	2x		
**Age:**	**Ø =** 24.5 years	Range = 21 years to 28 years		

Beginners group

IELY10
1.5 years, 2x week:
50 x 1,5 x 2 = **150**
Beginners
Male, 32 years

RABI25
1 Year, 1x week
1 x 50 x 1 = **50**
Beginners
Female, 23 years

RHBI13
2 years, 1x week
2 x 50 x 1 = **100**
Beginners
Female, 22 years

IAHE12
1.5 years, 2x week
1,5 x 50 x 2 = **150**
Beginners
Male, 33 years

TALÜ14
1.5 years, 1x week
1.5 x 50 x 1 = **75**
Beginners
Female, 24 years

IEBO03
4.5 years, 2x week
4.5 x 50 x 2 = **450**
Beginners
Female, 24 years

**Gender**	**Male**	**Female**			
	2x	4x			
**Experience in their sport**	**1 Jahr**	**1.5 years**	**2 years**	**2.5–3.5 years**	**4.5 years**
	1x	3x	1x	/	1x
**Training sessions per week in their sport**	**1x**	**2x**			
	3x	3x			
**Age:**	**Ø =** 26.33 years	Range = 22 years to 33 years			

Novices group

LIBI01
Soccer, 6 years, 3-4x week
Female, 23 years

REBI16
Swimming / Diving, 3 years, 1x week
Female, 24 years

REHA12
Soccer, 20 years, 3-4x week
Male, 25 years

ZAHO28
Handball 16 years, 3x week
Male, 21 years

IÜDA25
No Sport
Female, 19 years

HIDA14
No Sport
Female, 24 years

**Gender**	**Male**	**Female**	
	2x	4x	
**Sports**	**Team sport**	**Individual Sport**	**No Sport**
	3x	1x	2x
**Experience in their sport**	**1–5 years**	**5–10 years**	**>10 years**
	1x	1x	2x
**Training sessions per week in their sport**	**0–2**	**3–4**	**>4**
	3x	3x	/
**Age:**	**Ø =** 22.66 years	Range = 19 years to 25 years	
