# Supplementary material for: Expertise-dependent differences in mental representation metrics of pas de bourrée
Source: PLoS One. 2023 Oct 5;18(10):e0292133. doi: 10.1371/journal.pone.0292133 (PMC10553204; doi:10.1371/journal.pone.0292133)
Supplement: Attachment — Submitted filename: Response_to_reviewers_BS.pdf [file pone.0292133.s003.pdf]

Rebuttal letter of manuscript "Expertise-Dependent Differences in Mental Representation Metrics of pas de bourée" (PONE-D-23-11698)

Academic Editor

Dear Dr Makris,

Thank you very much for handling our manuscript and enabling us to provide a revised version. We have been committed to carefully considering all remarks to optimize the quality of the manuscript. Please find our responses to each of your remarks below**.**

1. **Please ensure that your manuscript meets PLOS ONE's style requirements, including those for file naming.**

   Authors' Response:

   Thank you for bringing this to our attention. We have revised the manuscript based on PLOS ONE's style requirements you attached and made the appropriate corrections in the manuscript. This includes corrections of the capitalization of the headings as well as the file naming for the figures.

2. **Please remove any funding-related text from the manuscript and let us know how you would like to update your Funding Statement. Please include your amended statements within your cover letter; we will change the online submission form on your behalf.**

   Authors' Response:

   We have removed the paragraph from the Acknowledgments Section, and we would kindly ask you to amend this correspondingly in our online submission by adding the following sentence to the Funding Statement:

   > "*We acknowledge support for the publication costs by the Open Access Publication Fund of Bielefeld University and the Deutsche Forschungsgemeinschaft (DFG). The funders had no role in study design, data collection and analysis, decision to publish, or preparation of the manuscript.*"

3. **Please include your full ethics statement in the 'Methods' section of your manuscript file. In your statement, please include the full name of the IRB or ethics committee who approved or waived your study, as well as whether or not you obtained informed written or verbal consent. If consent was waived for your study, please include this information in your statement as well.**

   Authors' Response:

   Thank you for pointing this out. We inserted the full name of the ethics committee of Bielefeld University, who gave written approval to our study, in the subsection "Execution" of the "Materials and methods" section (line 154), as follows:

"The study was approved by the Ethics Committee of Bielefeld University (EUB)."

4. **Please include captions for your Supporting Information files at the end of your manuscript, and update any in-text citations to match accordingly.**

   Authors' Response:

   Thank you for pointing this out. We have revised the captions for the supporting information files and provided in-text citations matching these captions (see lines 139 f., 152, 219 f., 339 f., 341 f.).

5. **Please review your reference list to ensure that it is complete and correct.**

   Authors' Response:

   We have reviewed our reference list for completeness and correctness. During the revision process, we added three new references into the text, as well as in the reference list. These are the following references:

(28.) Akoglu H. User's guide to correlation coefficients. Turkish Journal of Emergency Medicine. 2018;18(3):91–93. doi:https://doi.org/10.1016/j.tjem.2018.08.001.
(29.) Mayerl J, Fröhlich M, Pieter A, Kemmler W. In: Bivariate statistische Verfahren. Berlin, Heidelberg: Springer Berlin Heidelberg; 2020. p. 57–72. Available from: https://doi.org/10.1007/978-3-662-61039-8_6.
(30.) Alsaqr AM. Remarks on the use of Pearson's and Spearman's correlation coefficients in assessing relationships in ophthalmic data. African Vision and Eye Health. 2021;80(1):10. doi:https://doi.org/10.4102/aveh.v80i1.612.

(response to Reviewer 1 on next page)

Reviewer 1

Dear Dr. Wilkerson,

Thank you very much for taking the time to review our manuscript and for your appreciative and constructive comments. We found the advice helpful and understandable, and have incorporated the suggestions into our revision. We believe this improved the quality of the manuscript notably. Please find our responses to each of your remarks below**.**

1. **No mention of Institutional Review Board approval of the study procedures**

   Authors' Response:

   Thank you for pointing this out. We inserted the full name of the ethics committee of Bielefeld University, who gave written approval to our study, in the subsection "Execution" of the "Materials and methods" section (line 154), as follows:

   > *"The study was approved by the Ethics Committee of Bielefeld University (EUB)."*

2. **No mention of the manner in which participants were recruited/selected**

   Authors' Response:

   We included further information regarding the recruitment and selection of the participants in the subsection "Participants" of the "Materials and methods" section (line 137 f.), as follows:

   > *"In order to establish three perfectly balanced expertise groups of six participants each, a total of 18 participants aged between 19 and 33 years were recruited."*

   As well as in the "Execution" of the "Materials and methods" section (line 155 f.), as follows:

   > *"Participants were recruited in March 2021 at the campus of Bielefeld University and from a ballet school in Bielefeld (Germany), gave written informed consent to participate in the study..."*

3. **There is no information pertaining to power analysis for determination of the minimum number of participants need to avoid a Type II error. I do not consider power analysis to be an essential component of the research process, but some explanation is needed for the manner in which the 18 participants were recruited (i.e., 3 perfectly balanced groups of 6 participants each).**

   Authors' Response:

   Thank you very much for pointing out that we had not addressed this issue in our initial submission. We have performed a Power Analysis and provide detailed information on

the results in the revised subsection “Participants” of the “Materials and methods” section (lines 134 ff.), as follows:

> *“A power analysis with SPSS Version 29.0.0 estimated that a minimum sample size of 16 participants would have been necessary to determine significant Spearman correlation values of ρ = .7 or higher, which are commonly interpreted as “strong” [28–30], with at least 80% chance. In order to establish three perfectly balanced expertise groups of six participants each, a total of 18 participants aged between 19 and 33 years were recruited.”*

(response to Reviewer 2 on next page)

Reviewer 2:

Thank you very much for taking the time to review and for your commendatory and constructive comments upon our manuscript. We found the advice helpful and understandable, and have incorporated the suggestions into our revision. We believe this improved the quality of the manuscript notably. Please find our responses to each of your remarks below**.**

1. **Please provide sample size estimation in the manuscript.**

   Authors' Response:

   Thank you very much for pointing out that we had not addressed this issue in our initial submission. We have performed a Power Analysis and provide detailed information on the results in the revised subsection "Participants" of the "Materials and methods" section (lines 134 ff.), as follows:

   > *"A power analysis with SPSS Version 29.0.0 estimated that a minimum sample size of 16 participants would have been necessary to determine significant Spearman correlation values of ρ = .7 or higher, which are commonly interpreted as "strong" [28–30], with at least 80% chance. In order to establish three perfectly balanced expertise groups of six participants each, a total of 18 participants aged between 19 and 33 years were recruited."*

2. **considering the small sample size, this study should report as pilot study or preliminary study.**

   Authors' Response:

   We understand that the sample size, which was attained under constraints of the COVID-19 pandemic, may appear comparatively small. However, due to the fact that this participant number already exceeded the minimum sample size estimated by power analysis and yielded highly significant results, we tend to not classify the study as a mere pilot or preliminary study. Nevertheless, we agree that the sample size needs to be considered in interpreting the results of our study. Therefore, we have added a corresponding additional remark in the limitations of our study (lines 296 ff.):

   > *„Although the participant number exceeded the minimum sample size estimated by power analysis, future larger studies could facilitate the determination of more reliable estimates of the actual population values."*
